# Systemic pro-inflammatory cytokine status following therapeutic hypothermia in a piglet hypoxia-ischemia model

**DOI:** 10.1186/s12974-017-0821-x

**Published:** 2017-03-03

**Authors:** Eridan Rocha-Ferreira, Dorottya Kelen, Stuart Faulkner, Kevin D. Broad, Manigandan Chandrasekaran, Áron Kerenyi, Takenori Kato, Alan Bainbridge, Xavier Golay, Mark Sullivan, Boris W. Kramer, Nicola J. Robertson

**Affiliations:** 1 Institute for Women’s Health, University College London, 74 Huntley Street, London, WC1E 6AU UK; 2 First Department of Pediatrics, Semmelweis University, Budapest, Hungary; 3 Department of Medical Physics and Bioengineering, and Institute of Neurology, University College London, London, UK; 4 Institute of Neurology, University College London, London, UK; 5 Institute of Reproductive and Developmental Biology, Hammersmith Campus, Imperial College London, London, UK; 6 Institute of Oncology and Developmental Biology, Institute of Mental Health and Neuroscience, Maastricht University Medical Centre, Maastricht, The Netherlands

**Keywords:** Birth asphyxia, Cytokines, Biomarkers, Therapeutic hypothermia, Magnetic resonance spectroscopy, Neuroprotection

## Abstract

**Background:**

Inflammatory cytokines are implicated in the pathogenesis of perinatal hypoxia-ischemia (HI). The influence of hypothermia (HT) on cytokines after HI is unclear. Our aim was to assess in a piglet asphyxia model, under normothermic (NT) and HT conditions: (i) the evolution of serum cytokines over 48 h and (ii) cerebrospinal fluid (CSF) cytokine levels at 48 h; (iii) serum pro/anti-inflammatory cytokine profile over 48 h and (iv) relation between brain injury measured by magnetic resonance spectroscopy (MRS) and brain TUNEL positive cells with serum cytokines, serum pro/anti-inflammatory cytokines and CSF cytokines.

**Methods:**

Newborn piglets were randomized to NT (*n* = 5) or HT (*n* = 6) lasting 2–26 h after HI. Serum samples were obtained 4–6 h before, during and at 6–12 h intervals after HI; CSF was obtained at 48 h. Concentrations of interleukin (IL)-1β, −4, −6, −8, −10 and TNF-α were measured and pro/anti-inflammatory status compared between groups. White matter and thalamic voxel lactate/N-acetyl aspartate (Lac/NAA) (a measure of both oxidative metabolism and neuronal loss) were acquired at baseline, after HI and at 24 and 36 h.

**Results:**

Lac/NAA was reduced at 36 h with HT compared to NT (*p* = 0.013 basal ganglia and *p* = 0.033 white matter). HT showed lower serum TNF-α from baseline to 12 h (*p* < 0.05). Time-matched (acquired within 5 h of each other) serum cytokine and MRS showed correlations between Lac/NAA and serum IL-1β and IL-10 (all *p* < 0.01). The pro/anti-inflammatory ratios IL-1β/IL-10, IL-6/IL-10, IL-4/IL-10 and IL-8/IL-10 were similar in NT and HT groups until 36 h (24 h for IL-6/IL-10); after this, 36 h pro/anti-inflammatory cytokine ratios in the serum were higher in HT compared to NT (*p* < 0.05), indicating a pro-inflammatory cytokine surge after rewarming in the HT group. In the CSF at 48 h, IL-8 was lower in the HT group (*p* < 0.05). At 48 h, CSF TNF-α correlated with Lac/NAA (*p* = 0.02) and CSF IL-8 correlated with white matter TUNEL positive cell death (*p* = 0.04).

**Conclusions:**

Following cerebral HI, there was a systemic pro-inflammatory surge after rewarming in the HT group, which is counterintuitive to the putative neuroprotective effects of HT. While serum cytokines were variable, elevations in CSF inflammatory cytokines at 48 h were associated with MRS Lac/NAA and white matter cell death.

**Electronic supplementary material:**

The online version of this article (doi:10.1186/s12974-017-0821-x) contains supplementary material, which is available to authorized users.

## Background

Hypoxic-ischemic brain injury induces the release of cytokines and chemokines, which amplify pro-inflammatory cascades and recruit neutrophils and monocytes to areas of injury [1]. Over the hours and days after the injury, local and systemic production of cytokines recruit subsets of leukocytes to sites of CNS injury; this immune response is likely to influence the extent of secondary injury [2]. Indeed, recent successful therapeutic approaches for neuroprotection in the developing brain target recruitment of immune cells into the brain [3].

As neuroprotective therapies combined with hypothermia (HT) become a possibility for optimizing treatment of neonatal encephalopathy (NE) [4], early clinical bedside markers of injury severity are needed to allow treatments to be tailored to individual patients. Combinations of raised cytokines (e.g. cerebrospinal fluid (CSF) IL-6 and IL-8) have been shown to predict the severity NE [5]. Jenkins et al. showed that elevated serum levels of interleukin (IL)-6 and monocyte chemo-attractant protein (MCP)-1 from 0–9 h after perinatal hypoxia-ischemia (HI) are associated with non-survival of babies with NE, suggesting these might be important early biomarkers of futility of treatment or need for adjunct therapy [6]. However, the influence of HT on the pro/anti-inflammatory profile after HI is unknown. Recent studies suggest that HT has variable effects on inflammatory cytokines, depending on the site and timing of injury and whether there is co-existing infection [7, 8]. Although early peaks in IL-6 and monocyte MCP-1 within 0–9 h were predictive of adverse outcome, Jenkins et al. described a second peak in IL-6 and macrophage inflammatory protein (MIP)-1 alpha in HT patients who went on to have a good outcome [6]. These data suggest that cytokine levels may be specific to the phase of injury and repair and that cytokines may switch roles within a relatively short time after delivery.

Magnetic resonance spectroscopy (MRS) biomarkers correlate with injury severity after HI in the piglet asphyxia model [9, 10] and predict 1 year neurodevelopmental outcomes in babies who have suffered NE [11, 12]. MRS thalamic lactate/N-acetyl aspartate (Lac/NAA) is currently used as surrogate outcome measures in clinical neuroprotection studies of NE (https://www.npeu.ox.ac.uk/toby-xe). The Lac/NAA ratio reflects mitochondrial impairment and neuronal integrity; a high ratio in the first month after birth predicts a poor 12–18 month neurodevelopmental outcome [13, 14]. The ratios NAA/Cr will reflect mainly neuronal integrity and Lac/Cr or Lac/choline (Cho) mainly mitochondrial impairment. In a study looking at the associations between cytokines and MRS in NE, Bartha et al demonstrated that elevated IL-6, IL-1β, IL-8 and TNF-α in serum correlated with increased brain Lac/Cho ratios in the deep grey matter [15].

Clinical studies of cytokines can lead to variable results due to the variability in the timing of HI before birth, multiple organ involvement and the medical interventions that lead to a “cytokine storm”. Our aim was to use a preclinical piglet model which has a similar level of brain maturation to term human newborns, under normothermic (NT) and HT conditions with a standardized cerebral hypoxic-ischemic insult to assess (i) the evolution of serum cytokines (over 48 h) and (ii) CSF cytokine levels (at 48 h), (iii) pro- versus anti-inflammatory serum cytokine profiles over 48 h and (iv) the relation between brain injury severity measured by cerebral proton (^1^H) MRS and brain TUNEL positive cells with the cytokine concentrations in serum and CSF.

## Methods

### Animal experiments and surgical preparation

All animal experiments were carried out in accordance with the UK Home Office Guidelines – Animals (Scientific Procedures) Act 1986. Large white newborn male piglets aged <24 h (Table 1) were anesthetized and surgically prepared as described previously [16]. In brief, piglets were sedated with intramuscular midazolam (0.2 mg/kg), anesthetized with isoflurane (4% *v*/*v*) using a mask to facilitate tracheostomy and intubation and maintained (3% surgery, 2% otherwise). Mechanical ventilation was titrated to ensure partial arterial pressure of oxygen (PaO_2_) and carbon dioxide (PaCO_2_) were maintained at 8–13 and 4.5–6.5 kPa, respectively. An umbilical venous catheter was inserted to facilitate the infusion of maintenance fluids (10% dextrose, 60 ml/kg/day; morphine, 50 μg/kg/h) and antibiotics (benzylpenicilin, 50 mg/kg; gentamicin, 2.5 mg/kg every 12 h). An umbilical arterial catheter was inserted to facilitate continuous monitoring of heart rate (HR), mean arterial blood pressure measurement and arterial blood extraction to measure PaO_2_, PaCO_2_, pH, electrolytes, glucose (3–10 mmol/L) and lactate (I-Stat, Abbott Laboratories, Maidenhead, UK). Mean arterial blood pressure was maintained at approximately 40 mmHg using saline boluses, colloid (Gelofusin, B Braun Medical, Emmenbrucke, Switzerland) and infusions of inotropes (dopamine 5–20 μg/kg/min). Hyperglycemia (<10 mmol/L) was treated by substituting 10% with 5% dextrose; hyperglycemia (<20 mmol/L) was treated by substituting 5% dextrose for saline. Metabolic acidosis (base excess <−10) was corrected with sodium bicarbonate (8.4% *w*/*v*). All animals received continuous physiological monitoring (SA instruments, Stony Brook, NY) and extensive life support throughout experimentation. Arterial lines were maintained by infusing 0.9% saline solution (1 mL/h); heparin sodium (1 IU/mL) was added to prevent blockage. Both common carotid arteries were surgically isolated at the level of the fourth cervical vertebrae and encircled by remotely controlled vascular occluders (OC1.5 and OC2 mm, In Vitro Metric, Healdsberg, CA). After surgery, piglets were placed in a prone position in an acrylic pod with their heads immobilized.

**Table 1:** Physiological variables for piglets in NT and HT groups

Variables	Normothermia (NT) mean (SD)	Hypothermia (HT) mean (SD)	*p* Value
Post-natal age (h)	22.9 (0.9)	23.2 (0.8)	0.84
Body weight (g)	1583 (105)	1577 (91)	0.79
HR (bpm)			
Baseline	156 (12)	156 (12)	0.10
End of insult	219 (55)	199 (55)	0.06
2–26 h after time zero	162 (15)	132 (10)	0.10
48 h after time zero	127 (12)	157 (19)	0.02
MABP (mmHg)			
Baseline	46 (4)	43 (7)	0.69
End of insult	53 (8)	41 (6)	0.04
2–26 h after time zero	48 (3)	42 (3)	0.07
48 h after time zero	47 (11)	47 (10)	0.99
T rectal (°C)			
Baseline	38.8 (0.4)	38.7 (0.2)	0.82
End of insult	38.5 (0.4)	38.5 (0.3)	0.11
2–26 h after time zero	38.6 (0.1)	33.4 (0.2)	0.04
48 h after time zero	38.4 (0.4)	38.6 (0.5)	0.30
pH			
Baseline	7.49 (0.05)	7.50 (0.07)	0.39
Nadir of the insult	7.43(0.07)	7.49(0.11)	0.26
12 h after time zero	7.48(0.08)	7.48(0.16)	0.10
24 h after time zero	7.44(0.09)	7.47(0.11)	0.68
48 h after time zero	7.41(0.17)	7.46(0.11)	0.99
BE (mmol/L)			
Baseline	6.5 (3)	9.1 (2.4)	0.46
Nadir of the insult	1.3 (5.8)	4.5 (5)	0.16
12 h after time zero	5.6 (1.3)	7.6 (4.1)	0.99
24 h after time zero	4.9 (2)	7.5 (6.2)	0.63
48 h after time zero	0.7 (3.2)	3 (2.9)	0.29
Lactate (mmol/L)			
Baseline	2.7 (1.9)	2.3 (0.9)	0.84
Nadir of the insult	7.6 (2.9)	5.6 (2.1)	0.25
12 h after time zero	1.4 (0.3)	1.2 (0.5)	0.77
24 h after time zero	1.3 (0.5)	1.0 (0.3)	0.07
48 h after time zero	1.8 (0.9)	1.5 (0.4)	0.93
Glucose (mmol/L)			
Baseline	7.2 (1.1)	7.0 (1.0)	0.11
Nadir of the insult	9.0 (1.7)	8.5 (1.5)	0.36
12 h after time zero	5.6 (2.3)	8.5 (3.5)	0.39
24 h after time zero	4.6 (1.1)	5.2 (0.7)	0.39
48 h after time zero	5.4 (0.6)	7.0 (7.8)	0.04

Mean (SD) values are presented for each group. NT (*n* = 5) and HT (*n* = 6). Heart rate and blood pressure (mean arterial blood pressure; mmHg) data are omitted for 30 min after a saline/geoplasma bolus was administered or after cardiac arrest. Mann-Whitney analysis indicated the following in the HT group: a higher HR at 48 h (after rewarming), a lower MABP after HI (although still within the normal range), a lower temperature at 2–26 h (expected) and a higher blood glucose at 48 h

### Cerebral hypoxia-ischemia

A 65 × 55 mm, elliptical transmit/receive surface coil, tuned for ^31^P signal acquisition was secured to the head and the animal placed into the bore of a 9.4 Tesla Agilent spectrometer. ^31^P MRS data were acquired using a pulse-acquire sequence with a repetition time of 10 s and 6 averages; the acquisition time was 1 min. Transient HI was induced remotely by inflating simultaneously both vascular occluders and reducing the fractional inspired oxygen (FiO_2_) level to 12% (*v*/*v*). During HI, cerebral metabolism was monitored every minute by ^31^P-MRS, and β-nucleotide triphosphate (β-NTP; mainly ATP) peak height was continuously measured. Once the concentration of β-NTP declined to 40% of baseline, it was maintained at this value for 10 min through titration of the FiO_2_. Occluders were then deflated, F_i_O_2_ levels were normalized and ^31^P MRS spectra were acquired for a further 1 h to measure recovery from hypoxia-ischemia. The duration of the occlusion was 25–30 min. The combined hypoxic-ischemic insult and subsequent 60 min post-resuscitation period was used to measure the magnitude of acute energy deletion. This was quantified by measuring the time integral of the decrement of β-NTP/EPP [EPP=exchangeable phosphate pool=inorganic phosphate + phosphocreatine + (2*γ* + *β*)–NTP].

### Experimental groups

Following HI and resuscitation piglets were randomized into two groups, (i) normothermia (NT) (38.5 ± 0.5 °C throughout; *n* = 5) or (ii) 24 h whole body cooling 2–26 h HT (33.5 ± 0.5 °C; *n* = 6). We have previously shown this to be the optimal level of cooling in the piglet and corresponds to the body temperature in babies undergoing HT for NE [17, 18]. At 26 h after HI, cooled animals were rewarmed to NT at 0.5 °C/h. Forty-eight hours after resuscitation, piglets were euthanized with pentobarbital.

### ^1^H magnetic resonance spectroscopy

**Fig. 1:**
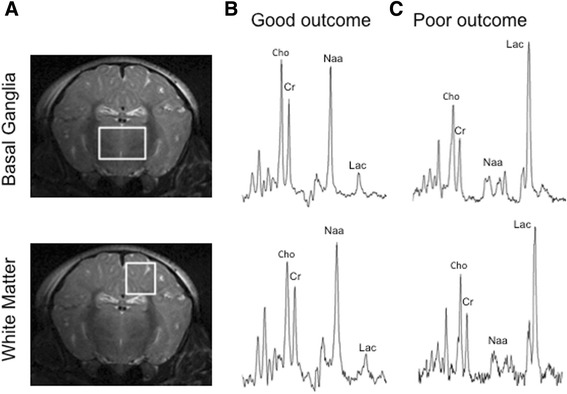
Piglet brain metabolic changes following hypoxia-ischemia injury. **a** Location of ^1^H MRS voxels superimposed on coronal 9.4 T brain MR images of a term piglet. The midparietal plane shows the location of the voxels on the basal ganglia and dorsal subcortical white matter forebrain regions. **b** Representative ^1^H MRS in the dorsal subcortical voxel of neonatal piglets with poor outcome 48 h after HI. Note the substantial increase in the lactate peak (Lac) and reduction of NAA. The creatine peak remains moderately unchanged. **c** Representative ^1^H MRS of a newborn piglet with normal neurodevelopmental outcome at 48 h of age. Note the absence of a lactate peak

^1^H MRS spectra were acquired using a 6.5 cm × 5.5 cm elliptical receive surface coil tuneable to ^1^H. Baseline spectra were acquired before HI and later at 24 and 48 h. LASER MRS [19] was acquired from two positions: deep grey matter centred on both lateral basal ganglia (15 × 15 × 10 mm voxel) and dorsal right subcortical white matter at the centrum semiovale level (white matter; 8 × 8 × 15 mm voxel). ^1^H MRS spectra were acquired with a repetition time of 5 s, 128 averages and an echo time of 288 ms (Fig. 1).

### Magnetic resonance spectroscopy analysis

^1^H MRS spectra were analyzed using AMARES [20] as implemented in the jMRUI software [21]. Relative signal amplitudes of total creatine, N-acetyl aspartate and lactate were measured for each spectrum. The ^1^H MRS outcome measures were Lac/Cr (measure of mitochondrial impairment), NAA/Cr (measure of neuronal integrity) and Lac/NAA (measure of both mitochondrial impairment and neuronal integrity). Metabolite peak/area under the curve ratios were plotted from baseline to 48 h post-insult for each voxel in each animal, on a logarithmic scale and analyzed according to the randomized group.

### Cytokine analysis

Blood samples were obtained from the umbilical artery immediately after surgery (baseline), during HI, at the point where β-NTP initially declined to 40% of baseline (NADIR), and at 6, 12, 24, 36 and 48 h after insult. Samples were centrifuged at 4000 rpm for 15 min and serum supernatant stored at −80 °C. CSF was obtained at 48 h post-insult by inserting a 21 gauge needle into the space between the 5th lumbar and 1st sacral vertebrae.

Cytokine concentrations were determined in standardized 50 μL samples using a fluorescence linked immunoabsorbant assay (Procarta Porcine Cytokine Assay Kits). Sensitivity and standard curve range were IL-1β (0.09 and 3.74–15300 pg/ml), IL-4 (0.072 and 1.44–5900 pg/mL), IL-6 (0.428 and 7.67–31400 pg/mL), IL-8 (1.420 and 15.77–64600 pg/mL), IL-10 (1.250 and 3.76–15400 pg/mL) and TNF-α (2.272 and 6.47–26500 pg/mL).

### Brain histology

At 48 h following HI, animals were euthanized with pentobarbital, and brains fixed through transcardial perfusion using 4% paraformaldehyde in PBS. After dissection, the brain was post-fixed for 7 days in 2% paraformaldehyde. Coronal 5-μm-thick sections of the right hemisphere, starting anterior to the optic chiasma, were embedded in paraffin wax. To assess extent of cell death, brain sections were stained for nuclear DNA fragmentation using histochemistry TUNEL assay as described previously [16]. Sections were dehydrated in xylene (3 × 10 min), rehydrated in decreasing concentrations of ethanol (100–70%) and followed by distilled water. To remove endogenous peroxidase, sections were pre-treated for 15 min in 3% H_2_O_2_ in methanol. This was followed by a 15 min peptidase predigestion with 20 μg/ml proteinase K (Promega) at 65 °C, and incubation for 2 h at 37 °C with TUNEL solution (Roche). Biotinylated dUTP was detected with avidin-biotinylated enzyme complex (Vector laboratories) and visualized using diaminobenzidine/H_2_O_2_ (Sigma) with CoCl_2_ and NiCl_2_ to intensify TUNEL histochemistry.

Double labelling of TUNEL and neurons (NeuN, 1:250; Millipore), astrocytes (GFAP, 1:5000; eBioscience), microglia (IBA-1, 1:100; Wako) and oligodendrocytes (Olig2, 1:500; Millipore) was performed by firstly staining the sections for TUNEL as described above, immediately followed by immunofluorescence for the aforementioned antibodies. In brief, sections were blocked with goat serum and incubated with primary antibody overnight at 4 °C. On the second day, sections were incubated with corresponding 488-conjugated secondary (1:200; Invitrogen) and tertiary (1:200, Invitrogen) antibodies, followed by Texas red streptavidin incubation (1:1000; Vector) and counterstained with DAPI (Vector).

TUNEL positive nuclei were quantified at ×40 magnification (sampling area of 0.76 mm^2^) by two independent investigators blinded to the experimental groups. This was performed in three non-overlapping fields per region per animal.

### Data analysis and statistics

For pairwise analysis of physiological data, the Kruskal-Walis rank test and Mann-Whitney test were used.

#### Effect of HT on Lac/NAA in basal ganglia and white matter

**Fig. 2:**
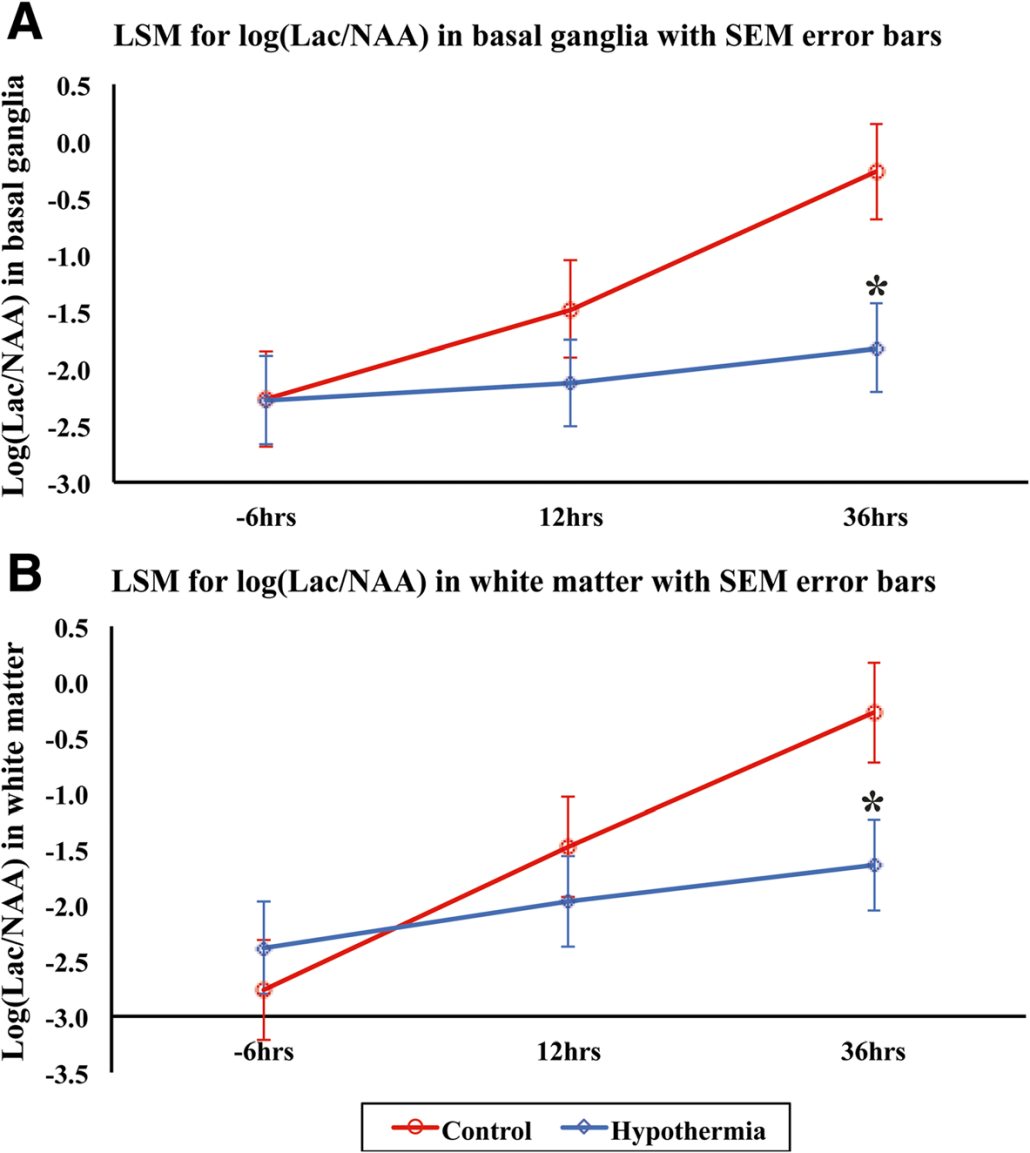
MRS log Lac/NAA at baseline, 24 and 36 h after HI for basal ganglia (**a**) and white matter (**b**). Least square mean (LSM) plots with standard error of the mean *error bars* for NT (*red*) and HT (*blue*) are shown. At 36 h, there was a significantly lower Lac/NAA in basal ganglia (*p* = 0.013) and white matter (*p* = 0.033) with HT compared to NT

All analyses were performed using the SAS JMP® v11.0.0 software. A statistical model was fitted to Lac/NAA. An analysis of variance (ANOVA) model was fitted, and the differences in the means on the log scale for the two treatment groups (HT versus NT) were estimated from the model at 36 h. The differences in treatment group least square means are shown graphically using SEM error bars (Fig. 2).

#### Evolution of serum cytokines over 48 h

Median serum interleukin (IL)-1β, IL-4, IL-6, IL-8, IL-10 and TNF-α concentrations were compared between NT and HT groups from baseline until termination (48 h) using a Mann-Whitney test.

#### Comparison of CSF cytokines at 48 h

Median serum interleukin (IL)-1β, IL-4, IL-6, IL-8, IL-10 and TNF-α concentrations were compared between NT and HT groups from baseline until termination (48 h) using a Mann-Whitney test.

#### Serum pro/anti-inflammatory cytokine profiles

Pro/anti-inflammatory cytokine ratios (IL-1β/IL-10, IL-6/IL-10, TNF-α/IL-10, IL-4/IL-10 and IL-8/IL-10) were calculated based on the serum data. Comparisons of these ratios between the NT and HT groups were made. All ratios were found to have positively skewed distributions, and thus, logs were calculated followed by ANOVA. Differences between means were calculated on the log scale and then back-transformed to original scale giving ratios of geometric means (with CI’s).

#### Relation between brain injury measured by magnetic resonance spectroscopy and brain TUNEL positive cells with cytokine concentrations in serum and CSF

MRS values were “matched” with serum cytokine measurements; MRS values within 5 h either side of the serum values were matched. This resulted in 49 matched values for the 11 animals. Statistical analysis of cytokine concentration employed a linear regression and one-way ANOVA, followed by Bonferroni correction. Analysis of Lac/NAA, NAA/Cr and Lac/Cr was performed using Spearman’s rank correlation. Assessment of the relation of TUNEL positive cells to cytokine levels at 48 h also used Spearman’s rank correlation. Results are presented as mean (±SD) unless stated otherwise, statistical significance was assumed for *p* < 0.05.

## Results

### Physiological measures

There were no intergroup differences in body weight, post-natal age, baseline physiological (heart rate and mean arterial blood pressure) and biochemical (blood lactate, base excess and glucose) measures at the beginning of experiment (Table 1). The hypoxic-ischemic insult severity was similar between both groups, with no mortality observed. In the HT compared to NT group, the heart rate was lower at 48 h (*p* = 0.02) and MABP lower just after the insult (*p* = 0.04). Fluid replacement was required for HT piglets—two animals required saline and four required treatment with dopamine (Table 2).

**Table 2:** Average total inotrope infusion and fluid replacement during 48 h after HI

Group	Mean (SD) use (mg/kg)	Median use (mg/kg)	Interquartile range (mg/kg)
Normothermia			
Dopamine	0 (0)	0	0
Saline bolus	0	0	0
Hypothermia			
Dopamine	9.70 (9.91)	8.3	12.86
Saline bolus	0	0	0

Median (IQR) values for inotrope infusion and saline bolus are presented for both groups. Mann-Whitney statistical assessment demonstrated no significant difference between normothermia and hypothermia groups for inotrope use and fluid replacement

### Reduced Lac/NAA at 48 h with HT

Lac/NAA was reduced at 36 h with HT compared to NT (*p* = 0.013 basal ganglia and *p* = 0.033 white matter) (Fig. 2).

### Evolution of serum cytokines

**Fig. 3:**
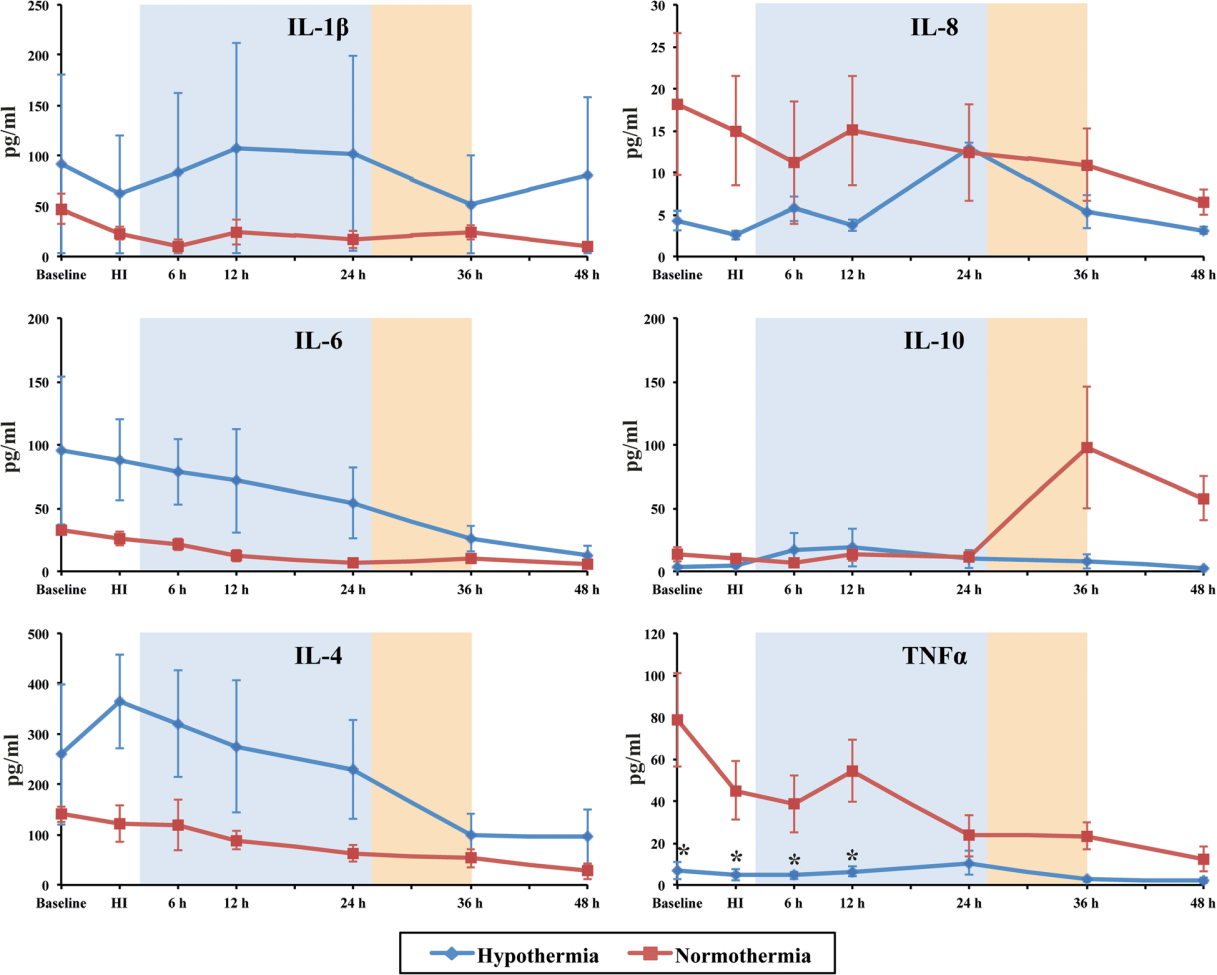
Median serum interleukin (IL)-1β, IL-4, IL-6, IL-8, IL-10 and TNF-α concentrations. Serum cytokine levels from both HT (*blue*) and NT (*red*) groups were assessed over time from baseline until termination (48 h). TNF-α levels were clearly decreased in the HT group between baseline and 12 h readouts, when compared to NT, *p < 0.05. The *shaded areas* represent the hypothermia (24 h, *blue*) and rewarming (10 h, *orange*) phases

**Fig. 4:**
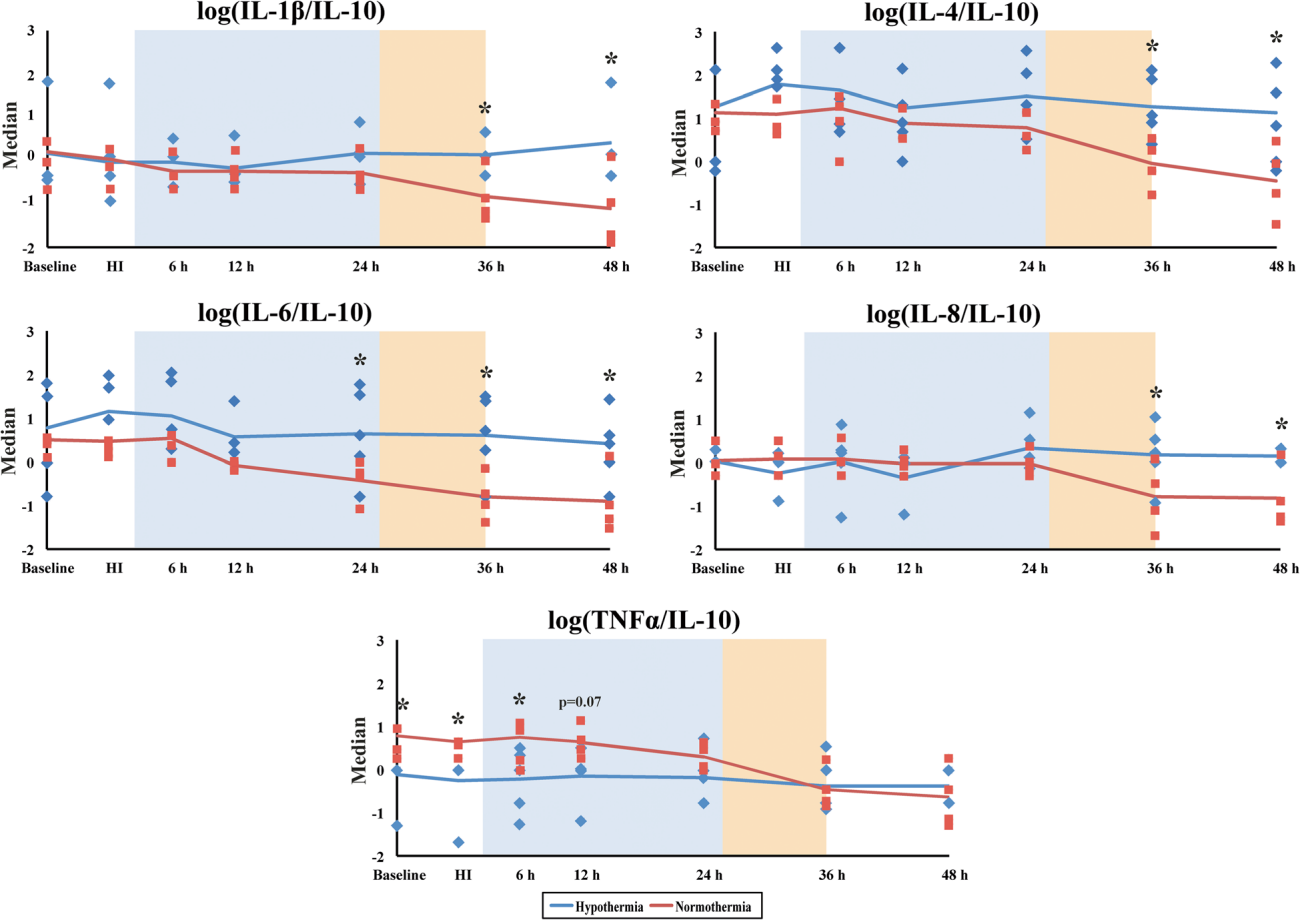
Pro/anti-inflammatory cytokine median log ratios (IL-1β/IL-10, IL-4/IL-10, IL-6/IL-10, IL-8/IL-10 and TNF-α/IL-10) are compared between the NT and HT groups from baseline to 48 h. All ratios were found to have positively skewed distributions, and thus, logs were calculated followed by ANOVA. For IL-1β/IL-10, IL-4/IL-10, IL-8/IL-10, there was a switch to a pro-inflammatory profile from 36 h; there was a switch to a pro-inflammatory profile from 24 h for IL-6/IL-10. For log TNF-α /IL-10, there was a switch from a pro-inflammatory state to neutral from 12 h after HI during HT. *p < 0.05. The *shaded areas* represent the hypothermia (24 h, *blue*) and rewarming (10 h, *orange*) phases

Individual cytokine analysis from baseline to 48 h post-HI showed a high degree of variability between groups (Fig. 3). Serum TNF-α levels were lower in the HT versus NT group at baseline, HI, 6 and 12 h after injury (*p* < 0.05). IL-10 increased after rewarming at 36 h in NT but not HT (Fig. 3). There were no significant differences between the other cytokines (Fig. 3). We calculated the pro/anti-inflammatory cytokine ratios of IL-1β/IL-10, IL-6/IL-10, TNF-α/IL-10, IL-4/IL-10 and IL8/IL-10 to assess the effect of HI and HT on the inflammatory status (Fig. 4 and Additional file 1: Table S1). After rewarming from 36 h after HI, there was a shift to a pro-inflammatory state with the following ratios becoming higher in the HT versus the NT group (*p* < 0.05 for all); IL-1β/IL-10, IL-6/IL-10 (higher in HT than NT from 48 h), IL-4/IL-10 and IL8/IL-10 (Fig. 4).

### Cerebrospinal fluid cytokines

**Fig. 5:**
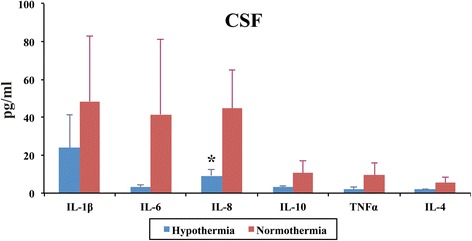
CSF IL-1β, IL-4, IL-6, IL-8, IL-10 and TNF-α concentrations 48 h after transient HI. HT resulted in visible reduction in overall CSF cytokine levels 48 h after HI. This reduction was significant for the IL-8 cytokine, *p < 0.05

CSF was collected 48 h after HI. In the HT group, lower concentrations of IL-8 only were seen in the CSF at 48 h (*p* < 0.05) (Fig. 5).

### Correlations between serum cytokines, CSF cytokines and pro/anti-inflammatory cytokine status with MRS and TUNEL positive cells

#### Serum cytokines

We assessed the relation between cytokine levels and Lac/NAA, Lac/Cr and NAA/Cr across both groups in all different time points. Serum IL-1β and IL-10 correlated positively with both basal ganglia (*p* < 0.001 each) and white matter Lac/NAA voxels (*p* = 0.003 and *p* = 0.001, respectively), as well as Lac/Cr basal ganglia (*p* < 0.001 and *p* = 0.003, respectively) and white matter (*p* = 0.005 and *p* = 0.001, respectively). There was a negative correlation between serum IL-1β and IL-10 and NAA/Cr in the basal ganglia (*p* = 0.02 and *p* = 0.01) and white matter (*p* = 0.004 and *p* = 0.005) voxels. There was a positive correlation for IL-8 and Lac/NAA in the white matter (*p* = 0.05). Correlations for other cytokines did not reach statistical significance and are shown in Table 3.

**Table 3:** Correlation of piglet neonatal serum cytokine levels with cerebral metabolite ratios assessed by proton-MRS

Cytokines	Lac/NAA				Naa/Cr				Lac/Cr			
	Basal ganglia		White matter		Basal ganglia		White matter		Basal ganglia		White matter	
	Coef	*p* Value	Coef	*p* Value	Coef	*p* Value	Coef	*p* Value	Coef	*p* Value	Coef	*p* Value
IL-1β	0.51	**<0.001**	0.42	**0.003**	−0.33	**0.02**	−0.31	**0.004**	0.51	**<0.001**	0.4	**0.005**
IL-4	−0.01	0.96	0.06	0.71	0.22	0.13	0.18	0.24	0.06	0.68	0.15	0.30
IL-6	0.02	0.87	0.15	0.30	0.18	0.21	0.06	0.71	0.09	0.55	0.22	0.14
IL-8	0.25	0.09	0.28	0.05	−0.18	0.21	−0.23	0.12	0.23	0.11	0.25	0.10
IL-10	0.46	**<0.001**	0.50	**<0.001**	−0.37	**0.01**	−0.40	**0.005**	0.42	**0.003**	0.46	**0.001**
TNF-α	0.13	0.38	0.09	0.53	0.02	0.88	−0.01	0.93	0.17	0.25	0.07	0.63

Bold *p* values indicate significance at *p* < 0.05

Statistical association based on Spearman’s rank correlation

#### CSF cytokines

CSF TNF-α levels at 48 h were positively correlated with white matter Lac/NAA and Lac/Cr (*p* = 0.02) acquired at 48 h. There was a negative correlation between TNF-α and NAA/Cr in basal ganglia (*p* = 0.003) and white matter (*p* = 0.005) voxels Table 4.

**Table 4:** Correlation of piglet neonatal CSF cytokine levels at 48 h after hypoxia-ischemia with cerebral metabolite ratios assessed by proton-MRS

Cytokines	Lac/NAA				NAA/Cr				Lac/Cr			
	Basal ganglia		White matter		Basal ganglia		White matter		Basal ganglia		White matter	
	Coef	*p* Value	Coef	*p* Value	Coef	*p* Value	Coef	*p* Value	Coef	*p* Value	Coef	*p* Value
IL-1β	0.59	0.13	0.49	0.22	−0.66	0.08	−0.50	0.20	0.59	0.13	0.49	0.22
IL-4	−0.06	0.88	0.09	0.83	0.17	0.67	−0.03	0.95	−0.06	0.88	0.09	0.83
IL-6	0.28	0.51	0.16	0.71	−0.16	0.71	−0.07	0.87	0.28	0.51	0.19	0.65
IL-8	−0.10	0.82	0.02	0.95	−0.05	0.91	0.10	0.82	−0.10	0.82	0.15	0.73
IL-10	0.15	0.73	0.16	0.70	−0.01	0.89	0.02	0.95	0.15	0.73	0.16	0.71
TNF-α	0.60	0.12	0.80	**0.02**	−0.89	**0.003**	−0.87	**0.005**	0.60	0.12	0.80	**0.02**

Bold *p* values indicate significance at *p* < 0.05

Statistical association based on Spearman’s rank correlation

#### TUNEL

**Fig. 6:**
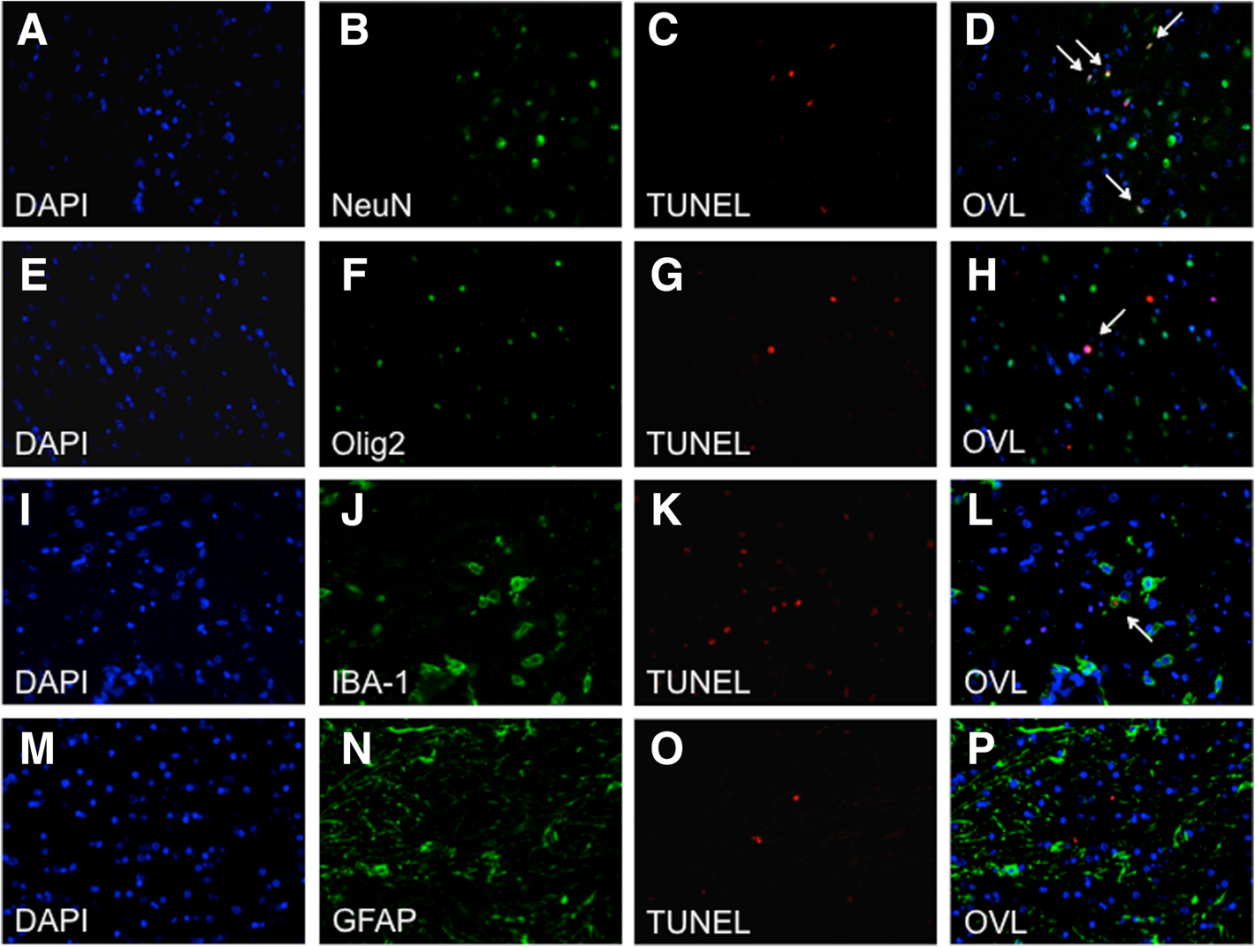
Hypoxia-ischemia causes predominantly neuronal cell death. Brain sections were stained with *DAPI* to fluorescently label nuclear DNA content (**a**, **e**, **i**, **m**) of different cell types: neurons (*NeuN*, **b**), oligodendrocytes (*Olig2*, **f**), microglia (*IBA-1*, **j**) and astroglial (*GFAP*, **n**), as well as *TUNEL* assay (**c**, **g**, **k**, **o**). Overlay (*OVL*) of images showed clear colocalization of *TUNEL* and neuronal cells (**d**, *white arrows*), with minimal colocalization with oligodendrocytes (**h**) and microglia (**l**). There was no evidence of *TUNEL* colocalization with astroglial (**p**) cells. Please note the border between the basal ganglia (*right*) and periventricular white matter (*left*) in 4. **d** Demonstrating clear presence of *TUNEL* positive cells in the grey matter

Assessment of TUNEL positive nuclei in the basal ganglia and 48 h cytokine levels (NT and HT groups together) showed a significant positive correlation with serum IL-1β (*p* < 0.004) and IL-8 (*p* < 0.005) (Table 5). CSF IL-8 was associated with white matter TUNEL positive cell death 48 h after HI (*p* = 0.04) (Table 5). In the basal ganglia voxel, immunofluorescent double staining demonstrated that NeuN (neurons) consistently colocalized with TUNEL positive cells (Fig. 6d). There was a minimal proportion of oligodendrocyte (Olig2) (Fig. 6h) and microglia (IBA-1) (Fig. 6l) colocalization with TUNEL cells, and no astroglial (GFAP) (Fig. 6p) co-labeling. In the white matter voxel, TUNEL positive cells colocalized with oligodendrocytes.

**Table 5:** Correlation of brain TUNEL positive cells with serum and CSF cytokine measurements

Measurements	Basal Ganglia		White matter	
	Coef	*p* Value	Coef	*p* Value
Serum cytokines				
IL-1β	0.92	**0.004**	0.56	0.19
IL-4	0.04	0.94	0.12	0.80
IL-6	0.00	1.00	0.05	0.91
IL-8	0.91	**0.005**	0.31	0.50
IL-10	0.44	0.33	−0.36	0.43
TNF-α	0.69	0.08	−0.12	0.79
CSF cytokines				
IL-1β	0.55	0.26	0.81	0.05
IL-4	0.34	0.51	−0.26	0.62
IL-6	−0.32	0.54	0.00	1.00
IL-8	−0.76	0.08	−0.83	**0.04**
IL-10	0.70	0.12	0.09	0.86
TNF-α	0.67	0.15	0.67	0.15

Bold *p* values indicate significance at *p* < 0.05

Association between the different variables was assessed based on Spearman’s rank correlation

#### Pro/anti-inflammatory cytokine status versus MRS and TUNEL

We assessed the relation between the inflammatory status and Lac/NAA in the basal ganglia and white matter. Apart from basal ganglia TNF-α/IL-10 which showed a weak correlation (*p* = 0.0537), there was no association between the pro/anti-inflammatory status and Lac/NAA (Additional file 2: Table S2). There was no relation between inflammatory status and TUNEL cell counts (Additional file 3: Table S3).

## Discussion

In this piglet model of perinatal HI, although we found high variability in serum cytokine concentrations at baseline, we saw a switch to a pro-inflammatory state after rewarming in the HT group. We saw an increase in the following ratios following rewarming at 36 h; IL-1β/IL-10, IL-4/IL-10 and IL8/IL-10. We saw an increase in IL-6/IL-10 at the end of HT from 24–48 h. There was a pro-inflammatory state with rewarming after HT. In the CSF at 48 h, HT was associated with lower levels of IL-8. Brain injury as measured by MRS and TUNEL positive cells was associated with some pro-inflammatory cytokines (IL-1β, IL-8) but the pro/anti-inflammatory ratios were not associated with Lac/NAA (mitochondrial impairment) apart from a weak correlation with TNF-α/IL-10.

Increased serum concentrations of IL-1β, IL-6 and TNF-α have been found in term infants diagnosed with CP compared to term infants with normal neurological outcomes [22]. TNF-α has been associated with impaired neurological outcomes following HI [15, 23, 24]. The precise effects of HT on the pro/anti-inflammatory state after HI are unknown and is likely to be dependent on the phase of injury and endogenous repair. HT is now standard clinical care for babies with moderate to severe NE in the UK and developed world [25]. There are however around 50% of infants who, despite treatment, have adverse neurodevelopmental outcomes. It is unclear whether this partial treatment effect is related to severity, type, or timing of injury in relation to cooling or accompanying infection. The neuroprotective effect of HT is thought to be due to a reduction in brain metabolism and preservation of ATP [26] and reducing apoptotic cascades [27]; the effect of HT on neuroinflammatory cascades is still unclear however. In a recent piglet study, immediate hypothermia after HI was associated with an early reduction in brain TNF-α at 6 h [28]. Other studies suggest that HT does not reduce the pro-inflammatory state. An in vitro study on the effect of temperature on cytokine release from microglia showed that IL-10 was down-regulated by HT; this suggests a pro- rather than anti-inflammatory effect of HT [29]. In a rat, pre-clinical model of inflammatory sensitized HIE, neuroprotection from HT was independent of the interleukin-1 system and was dependent on an increase in antioxidant enzymes [30]. Rapid rewarming (by 3 °C over 20 min) has been associated with a pro-inflammatory state in a rodent model, with a robust increase in IL-6 and IL-1β [31]. In our study, we rewarmed at a rate of 0.5 °C/h, which is similar to clinical protocols of 0.5 °C over 1–2 h.

While our data indicate a systemic pro-inflammatory effect of HT, HT showed significantly decreased levels of CSF IL-8 at 48 h. HT showed decreased CSF cytokine levels overall with significance for IL-8. We saw that increased CSF IL-8 was highly associated with TUNEL positive cell death in the white matter. Our lower CSF IL-8 at 48 h with HT could reflect inhibition of NF-kB activation and decreased microglial production of IL-10, TNF-α and nitric oxide synthases, interferon gamma, IL-2, IL-1β and MIP in the brain [29, 32].

Our data are also consistent with accumulating evidence of different roles that cytokines may have after HI. Cytokine and chemokine actions may be specific to the phase of injury and recovery, switching roles within a relatively short time after HI. Although IL-6 has some known anti-inflammatory properties, it has been associated with pro-inflammatory outcomes and worsening outcomes following birth asphyxia. CSF IL-6 and TNF-α have both shown a direct association with degree of NE, and IL-6 has been shown to predict neurodevelopmental outcome [33]. In another study, CSF IL-6, IL-1β and TNF-α concentrations were significantly increased in NE infants compared to healthy controls [23]. Both studies demonstrated a high CSF/serum ratio, suggesting cytokine production in the brain in addition to the systemic cytokines crossing the blood-brain barrier [23, 33]. However, in a study of adults with stroke and no signs and symptoms of concomitant infection, IL-6 showed a significant inverse correlation with final neurological impairment and infarct size [34]. Indeed IL-6 has been seen to have neurotrophic and anti-inflammatory effects [35]; in the stroke study IL-6 and TNF-α were inversely correlated, suggesting IL-6 might counterbalance the potential detrimental effects of TNF-α [34]. In our study, there was an increased TNF-α/IL-10 in the HT group over the first 12 h but no difference after this point. Jenkins studied the cytokine profiles in babies with NE randomized to either HT or NT. In HT babies, there was a biphasic pattern of IL-6 and IL-8 with an early and delayed peak; the delayed peak occurred between 24 and 56 h only in the HT babies. IL-6 may therefore be a subacute marker of patients who have activated important mechanisms of repair and consequently will have better outcomes than those who do not have a secondary peak. In an in vitro study of astrocytes exposed to hypoxia and re-oxygenation, enhanced transcription and release of IL-6 was observed after the burst of reactive oxygen species; survival of PC12 cells was improved when exposed to this medium and blocked with neutralizing anti IL-6 antibody [36]. Further possible beneficial effects of IL-6 have been described and include decreased glutamate toxicity, increased JAK-STAT3 signalling and induction of anti apoptotic Bcl proteins [37, 38]. We also saw an increase in IL-8/IL-10 in the HT group; IL-8 has been shown to stimulate angiogenesis. Taken together, the switch to a pro/anti-inflammatory state from 24–36 h after HT may be a marker of endogenous regeneration and repair.

We studied the association between serum and CSF cytokine levels and mitochondrial impairment using MRS biomarkers which correlate with injury severity after HI in the piglet [10] and outcome in NE [11, 12]. Lac/NAA has been used as a surrogate outcome measures in clinical neuroprotection studies of NE (https://www.npeu.ox.ac.uk/toby-xe). High levels of Lac/NAA, Lac/Cr and low NAA/Cr on MRS in neonates in the first month after birth are predictive of poor 12–18 month neurodevelopmental outcome [11, 13, 14]. We identified a significant positive correlation between higher serum IL-1β and IL-10 cytokines and Lac/NAA in basal ganglia and white matter voxels. In the CSF, we observed a positive correlation between TNF-α levels, Lac/NAA and Lac/Cr in the white matter and a significant negative correlation to NAA/Cr in both voxels. We did not see any relation between the pro/anti-inflammatory cytokine ratios and CSF cytokines or TUNEL positive cells; this may be due to the complex effects of cytokines at the different phases of injury and the single time point of study.

Our study has some limitations. Baseline levels of serum cytokines varied between piglets, and it is possible that this could affect the validity of our comparison; however, this is likely to reflect the situation in human neonates. Importantly, we saw a raised TNF-α/IL-10 from baseline to 12 h in the HT group; this was unexplained and could influence the response to HT. Mechanical ventilation was started prior to surgery; this could influence cytokine levels in the blood. Rodent studies have shown elevated CXCL-2 and IL-6 mRNA levels [39] and increased mRNA levels of MIP-2 and IL-10 with ventilation [40]. In a similar way to the clinical HT trials, we observed reduced heart rate, bradycardia and hypotension requiring inotropic support [18, 41, 42]. Four out of six HT animals required dopamine to support the blood pressure. Dopamine in itself can increase secretion of IL-10 and/or TNF-α by normal resting T cells [43]. However, despite dopamine treatment, serum levels of IL-10 and TNF-α remained low throughout the study.

## Conclusions

Following cerebral HI, there was a systemic pro-inflammatory surge after rewarming in the HT group, which is counterintuitive to the putative neuroprotective effects of HT. While serum cytokines were variable, elevations in CSF inflammatory cytokines at 48 h were associated with MRS Lac/NAA and white matter cell death. The role of cytokines during and after cooling may change, and further work is needed to explore whether HT should be complemented with anti-inflammatory therapies for maximal benefit. We show an association between serum IL-1β, IL-10, CSF TNF-α and MRS Lac/NAA thus relating mitochondrial impairment and neuronal integrity with pro-inflammatory cytokines. The reduced CSF IL-8 at 48 h may reflect a mechanism or effect of decreased injury with HT.

## Additional files

Additional file 1: Table S1. Pro/anti-inflammatory cytokine ratios (log10 and original scale) in NT and HT groups from baseline to 48 h. (DOCX 22 kb)
Additional file 2: Table S2. Cytokine pro/anti-inflammatory ratios versus log Lac/NAA in basal ganglia and white matter. (DOCX 14 kb)
Additional file 3: Table S3. Pro/anti-inflammatory cytokine ratios versus mean TUNEL counts. (DOCX 14 kb)

## Abbreviations

CSF: Cerebrospinal fluid
EPP: Exchangeable phosphate pool
FiO2: Fractional inspired oxygen
HR: GFAP, glial fibrillary acidic protein heart rate
IBA-1: Ionized calcium-binding adapter molecule 1
IL: Interleukin
Lac/NAA: Lactate/N-acetyl aspartate
MCP-1: Monocyte chemoattractant protein-1
MIP-1: Macrophage inflammatory protein-1
MRS: Magnetic resonance spectroscopy
Naa/Cr: N-acetyl aspartate/creatine
NE: Neonatal encephalopathy
Olig2: Oligodendrocyte transcription factor
OLV: Overlay
PaCO_2_: Partial arterial carbon dioxide
PaO_2_: Partial arterial pressure of oxygen
SD: Standard deviation
TNF-α: Tumour necrosis factor alpha
β-NTP: β-nucleotide triphosphate
