# Supplementary material for: Systemic pro-inflammatory cytokine status following therapeutic hypothermia in a piglet hypoxia-ischemia model
Source: J Neuroinflammation. 2017 Mar 3;14:44. doi: 10.1186/s12974-017-0821-x (PMC5335722; doi:10.1186/s12974-017-0821-x)
Supplement: Additional file 1: Table S1. — Pro/anti-inflammatory cytokine ratios (log10 and original scale) in NT and HT groups from baseline to 48 h. (DOCX 22 kb) [file 12974_2017_821_MOESM1_ESM.docx]

| **Additional file 1: Table S1 Pro/anti inflammatory cytokine ratios (log10 and original scale) in NT and HT groups from baseline to 48h** | | | | | | | | | | | | | | | | | | | |
| --- | --- | --- | --- | --- | --- | --- | --- | --- | --- | --- | --- | --- | --- | --- | --- | --- | --- | --- | --- |
|  | |  | |  | | |  |  | |  | | |  |  | | |  |  | |
|  |  | | **Analysis on Log10 Scale** | | | | | | | | | **Analysis on Original Scale** | | | | | | |  |
| **Ratio** | **Time (h)** | | **Difference in means on log10 Scale (Hypo - Normo)** | | **Std Err Dif** | **95% Lower CL for Difference** | | | **95% Upper CL for Difference** | | **p-Value** | **Ratio of Geometric Means (Hypo/Normo)** | | | **95% Lower CL for Geom Mean Ratio** | **95% Upper CL for Geom Mean Ratio** | | |  |
| log(IL-1β/IL-10) | -6 | | -0.01 | | 0.44 | -0.89 | | | 0.87 | | 0.986 | 1.0 | | | 0.1 | 7.4 | | |  |
| log(IL-1β/IL-10) | 0 | | -0.05 | | 0.41 | -0.88 | | | 0.79 | | 0.914 | 0.9 | | | 0.1 | 6.1 | | |  |
| log(IL-1β/IL-10) | 6 | | 0.20 | | 0.45 | -0.71 | | | 1.10 | | 0.667 | 1.6 | | | 0.2 | 12.6 | | |  |
| log(IL-1β/IL-10) | 12 | | 0.05 | | 0.44 | -0.82 | | | 0.93 | | 0.902 | 1.1 | | | 0.1 | 8.5 | | |  |
| log(IL-1β/IL-10) | 24 | | 0.40 | | 0.41 | -0.43 | | | 1.24 | | 0.333 | 2.5 | | | 0.4 | 17.3 | | |  |
| log(IL-1β/IL-10) | 36 | | 0.87 | | 0.41 | 0.04 | | | 1.70 | | **0.041** | 7.4 | | | 1.1 | 50.4 | | |  |
| log(IL-1β/IL-10) | 48 | | 1.37 | | 0.44 | 0.50 | | | 2.25 | | **0.003** | 23.7 | | | 3.1 | 178.8 | | |  |
| log(IL-6/IL-10) | -6 | | 0.27 | | 0.52 | -0.78 | | | 1.31 | | 0.609 | 1.9 | | | 0.2 | 20.6 | | |  |
| log(IL-6/IL-10) | 0 | | 0.67 | | 0.49 | -0.32 | | | 1.66 | | 0.179 | 4.7 | | | 0.5 | 46.1 | | |  |
| log(IL-6/IL-10) | 6 | | 0.53 | | 0.54 | -0.55 | | | 1.61 | | 0.332 | 3.4 | | | 0.3 | 40.4 | | |  |
| log(IL-6/IL-10) | 12 | | 0.64 | | 0.52 | -0.40 | | | 1.69 | | 0.223 | 4.4 | | | 0.4 | 48.7 | | |  |
| log(IL-6/IL-10) | 24 | | 1.06 | | 0.49 | 0.07 | | | 2.05 | | **0.037** | 11.4 | | | 1.2 | 112.2 | | |  |
| log(IL-6/IL-10) | 36 | | 1.42 | | 0.49 | 0.43 | | | 2.41 | | **0.006** | 26.4 | | | 2.7 | 258.9 | | |  |
| log(IL-6/IL-10) | 48 | | 1.33 | | 0.52 | 0.28 | | | 2.37 | | **0.014** | 21.3 | | | 1.9 | 236.7 | | |  |
| log(TNFα/IL-10) | -6 | | -0.89 | | 0.43 | -1.76 | | | -0.01 | | **0.047** | 0.1 | | | 0.0 | 1.0 | | |  |
| log(TNFα/IL-10) | 0 | | -0.93 | | 0.41 | -1.76 | | | -0.10 | | **0.029** | 0.1 | | | 0.0 | 0.8 | | |  |
| log(TNFα/IL-10) | 6 | | -0.97 | | 0.45 | -1.87 | | | -0.06 | | **0.036** | 0.1 | | | 0.0 | 0.9 | | |  |
| log(TNFα/IL-10) | 12 | | -0.80 | | 0.43 | -1.67 | | | 0.08 | | 0.074 | 0.2 | | | 0.0 | 1.2 | | |  |
| log(TNFα/IL-10) | 24 | | -0.49 | | 0.41 | -1.32 | | | 0.34 | | 0.238 | 0.3 | | | 0.0 | 2.2 | | |  |
| log(TNFα/IL-10) | 36 | | 0.07 | | 0.41 | -0.76 | | | 0.90 | | 0.863 | 1.2 | | | 0.2 | 8.0 | | |  |
| log(TNFα/IL-10) | 48 | | 0.27 | | 0.43 | -0.61 | | | 1.14 | | 0.538 | 1.9 | | | 0.2 | 13.9 | | |  |
| log(IL-4/IL-10) | -6 | | 0.14 | | 0.52 | -0.91 | | | 1.18 | | 0.795 | 1.4 | | | 0.1 | 15.2 | | |  |
| log(IL-4/IL-10) | 0 | | 0.67 | | 0.49 | -0.32 | | | 1.66 | | 0.181 | 4.7 | | | 0.5 | 46.0 | | |  |
| log(IL-4/IL-10) | 6 | | 0.41 | | 0.54 | -0.67 | | | 1.49 | | 0.449 | 2.6 | | | 0.2 | 30.9 | | |  |
| log(IL-4/IL-10) | 12 | | 0.35 | | 0.52 | -0.69 | | | 1.40 | | 0.502 | 2.2 | | | 0.2 | 25.0 | | |  |
| log(IL-4/IL-10) | 24 | | 0.71 | | 0.49 | -0.28 | | | 1.71 | | 0.155 | 5.2 | | | 0.5 | 50.9 | | |  |
| log(IL-4/IL-10) | 36 | | 1.31 | | 0.49 | 0.31 | | | 2.30 | | **0.011** | 20.3 | | | 2.1 | 199.6 | | |  |
| log(IL-4/IL-10) | 48 | | 1.57 | | 0.52 | 0.52 | | | 2.61 | | **0.004** | 36.8 | | | 3.3 | 409.8 | | |  |
| log(IL-8/IL-10) | -6 | | -0.01 | | 0.37 | -0.76 | | | 0.73 | | 0.976 | 1.0 | | | 0.2 | 5.4 | | |  |
| log(IL-8/IL-10) | 0 | | -0.33 | | 0.35 | -1.04 | | | 0.38 | | 0.352 | 0.5 | | | 0.1 | 2.4 | | |  |
| log(IL-8/IL-10) | 6 | | -0.07 | | 0.38 | -0.84 | | | 0.70 | | 0.862 | 0.9 | | | 0.1 | 5.0 | | |  |
| log(IL-8/IL-10) | 12 | | -0.33 | | 0.37 | -1.07 | | | 0.42 | | 0.381 | 0.5 | | | 0.1 | 2.6 | | |  |
| log(IL-8/IL-10) | 24 | | 0.36 | | 0.35 | -0.34 | | | 1.07 | | 0.305 | 2.3 | | | 0.5 | 11.8 | | |  |
| log(IL-8/IL-10) | 36 | | 0.97 | | 0.35 | 0.26 | | | 1.67 | | **0.009** | 9.3 | | | 1.8 | 47.3 | | |  |
| log(IL-8/IL-10) | 48 | | 0.97 | | 0.37 | 0.23 | | | 1.72 | | **0.012** | 9.4 | | | 1.7 | 52.5 | | |  |
