# Supplementary material for: Systemic pro-inflammatory cytokine status following therapeutic hypothermia in a piglet hypoxia-ischemia model
Source: J Neuroinflammation. 2017 Mar 3;14:44. doi: 10.1186/s12974-017-0821-x (PMC5335722; doi:10.1186/s12974-017-0821-x)
Supplement: Additional file 2: Table S2. — Cytokine pro/anti-inflammatory ratios versus log Lac/NAA in basal ganglia and white matter. (DOCX 14 kb) [file 12974_2017_821_MOESM2_ESM.docx]

| **Variable** | **by Variable** | **Correlation** | **Count** | **Lower 95%** | **Upper 95%** | **Signif Prob** |
| --- | --- | --- | --- | --- | --- | --- |
| **IL-1β/IL-10** | log(Lac/NAA) in bg | 0.71 | 6 | -0.24 | 0.97 | 0.1123 |
| **IL-6/IL-10** | log(Lac/NAA) in bg | -0.32 | 6 | -0.90 | 0.66 | 0.5381 |
| **TNFα/IL-10** | log(Lac/NAA) in bg | 0.80 | 6 | -0.02 | 0.98 | **0.0537** |
| **IL-4/IL-10** | log(Lac/NAA) in bg | -0.27 | 6 | -0.89 | 0.70 | 0.611 |
| **IL-8/IL-10** | log(Lac/NAA) in bg | -0.06 | 6 | -0.83 | 0.79 | 0.9152 |
| **IL-1β/IL-10** | log(Lac/NAA) in wm | -0.48 | 5 | -0.96 | 0.69 | 0.4077 |
| **IL-6/IL-10** | log(Lac/NAA) in wm | -0.44 | 5 | -0.95 | 0.72 | 0.4616 |
| **TNFα/IL-10** | log(Lac/NAA) in wm | 0.52 | 5 | -0.67 | 0.96 | 0.3707 |
| **IL-4/IL-10** | log(Lac/NAA) in wm | -0.41 | 5 | -0.95 | 0.74 | 0.4962 |
| **IL-8/IL-10** | log(Lac/NAA) in wm | -0.58 | 5 | -0.97 | 0.62 | 0.3025 |

**Additional file 1: Table S2 Cytokine Pro/anti inflammatory ratios versus log Lac/NAA in basal ganglia and white matter**

**There was a weak correlation only between TNFα/IL-10 and Lac/NAA in the basal ganglia**
