# Supplementary material for: Systemic pro-inflammatory cytokine status following therapeutic hypothermia in a piglet hypoxia-ischemia model
Source: J Neuroinflammation. 2017 Mar 3;14:44. doi: 10.1186/s12974-017-0821-x (PMC5335722; doi:10.1186/s12974-017-0821-x)
Supplement: Additional file 3: Table S3. — Pro/anti-inflammatory cytokine ratios versus mean TUNEL counts. (DOCX 14 kb) [file 12974_2017_821_MOESM3_ESM.docx]

| **Variable** | **by Variable** | **Correlation** | **Count** | **Lower 95%** | **Upper 95%** | **Signif Prob** |
| --- | --- | --- | --- | --- | --- | --- |
| **IL-1β/IL-10** | Mean TUNEL | 0.73 | 6 | -0.20 | 0.97 | 0.1005 |
| **IL-6/IL-10** | Mean TUNEL | 0.50 | 6 | -0.52 | 0.93 | 0.3103 |
| **TNFα/IL-10** | Mean TUNEL | 0.78 | 6 | -0.08 | 0.97 | **0.0664** |
| **IL-4/IL-10** | Mean TUNEL | 0.69 | 6 | -0.28 | 0.96 | 0.1315 |
| **IL-8/IL-10** | Mean TUNEL | 0.17 | 6 | -0.74 | 0.86 | 0.7454 |

**Additional file 1: Table S3 Pro/anti inflammatory cytokine ratios versus mean TUNEL counts**

**There was a weak correlation between TNFα/IL-10 and mean TUNEL counts**
